# “We give water or porridge, but we don’t really know what the child wants:” a qualitative study on women’s perceptions and practises regarding exclusive breastfeeding in Kilimanjaro region, Tanzania

**DOI:** 10.1186/s12884-018-1962-3

**Published:** 2018-08-08

**Authors:** Melina Mgongo, Tamara H. Hussein, Babill Stray-Pedersen, Siri Vangen, Sia E. Msuya, Margareta Wandel

**Affiliations:** 1 Institute of Clinical Medicine, University of Oslo, Oslo, Norway; 2 Better Health for African Mother and Child, P.O. Box 8418, Moshi, Tanzania; 3 Department of Nutrition, Institute of Basic Medical Sciences, University of Oslo, Oslo, Norway; 4 Division of Gynaecology and Obstetrics, Oslo University Hospital, Rikshospitalet, Oslo, Norway; 5 Institute of Public Health, Department of Epidemiology and Biostatistics, Kilimanjaro Christian Medical University College (KCMUCo), P.O. Box 2240, Moshi, Tanzania; 6 Department of Community Medicine, Kilimanjaro Christian Medical Centre (KCMC), P.O. Box 3010, Moshi, Tanzania; 7 Norwegian National Advisory Unit for Women’s health, Oslo, Norway

**Keywords:** Exclusive breastfeeding, Breastfeeding knowledge, Breastfeeding practise, Kilimanjaro, Tanzania

## Abstract

**Background:**

World Health Organization (WHO) recommends exclusive breastfeeding (EBF) as the optimal way to feed infants below 6 months of age. The benefits of EBF are well documented. However, in Tanzania, EBF is still rarely practised. This study explored the knowledge, attitudes and practises of EBF among mothers in Kilimanjaro region of northern Tanzania.

**Methods:**

This is a qualitative research study. The three districts in Kilimanjaro region namely Same, Moshi Municipal Council and Rombo districts were selected. In each district, three focus group discussions (FGDs) with mothers of infants aged 0–12 months were conducted. A total of 78 mothers participated in the focus group discussion.

**Results:**

The main result is that most of the mothers had a theoretical knowledge of the benefits of EBF but were not able to practise this knowledge for a range of reasons. The reasons for not practising EBF in real life included poor maternal nutrition, the pressure for women to return to work, inadequate knowledge about expressing breast milk, and perceived insufficiency of milk supply. Additionally, mothers received conflicting advice from a range of sources including close relatives, community members and health care providers, and they often choose the advice of their elders. Mothers also offered suggestions on ways to improve EBF including educating the community on the benefits of EBF.

**Conclusion:**

The results show that the women need support from close relatives and employers to successfully practise EBF. This presents a need for involving close relatives in EBF interventions, as they are important sources of breastfeeding information in the community**.** Additionally, behavioural interventions that promote optimal breastfeeding practises might help to improve exclusive breastfeeding.

## Background

The World Health Organization (WHO) recommends exclusive breastfeeding as an optimal way to feed infants [1]. It is the most effective intervention to improve child health and survival, especially in developing countries [2]. Exclusive breastfeeding (EBF) is defined as feeding the infant with breast milk alone for the first 6 months of life. The infant is allowed to take drops of vitamins, minerals and Oral Rehydration Solution, if prescribed [3]. EBF has been documented to confer many benefits to the child and the mother. For the child, breast milk is highly nutritious and contains antibodies that protect the child against diseases [1, 2, 4]. Children who are exclusively breastfed have decreased risk of getting infections and reduced risks of chronic illnesses later in life [5–7]. Furthermore, exclusively breastfed children have been shown to have good sensory and cognitive development, less risk of being malnourished and decreased risk of mortality and morbidity [2, 8]. For the mother, initiation of breastfeeding helps uterine contractions for easy removal of placenta and EBF has a hormonal effect which helps to delay menstruation. Longer duration of breastfeeding is associated with decreased risks of ovarian and breast cancer [1, 9].

Despite the well documented benefits of EBF, globally only 43% of infants are exclusively breastfed [10]. In developing countries the rate is lower at 37% [9]. In Sub Saharan Africa (SSA), a region with high rates of child mortality and malnutrition, only 36% of infants are exclusively breastfed [7, 11].

WHO has developed various strategies to promote the practise of EBF globally. Tanzania has adopted various WHO strategies to promote EBF practise in the country. The strategies include infant and young child feeding (IYCF), the Baby Friendly Hospital Initiative (BFHI) and Prevention of Mother to Child Transmission of HIV (pMTCT) [1, 12]. However, there has been a slow increase in the rates of EBF in the country. According to the Tanzania Demographic and Health Survey, only 59% of infants are exclusively breastfed and the rates of initiation of breastfeeding is still low. The country still faces the challenges of high infant mortality rate (67/1000 live births) and high stunting (34%) with the main causes being malnutrition and infections [13]. In Kilimanjaro region, a previous study showed that only about 29% of infants aged 0–5 months were exclusively breastfed [14]. This indicates that a large proportion of infants are introduced to complementary feeding before reaching 6 months of age.

Different studies have been conducted to assess the factors that influence EBF practise in Tanzania. These studies used quantitative methods of data collection and found that the factors that influenced the EBF practise include residence, maternal education, child age, counselling on breastfeeding, assistance by health care provider and delivery in the baby friendly health facility [14–17]. However there are limited qualitative studies that have explored the reasons for the low practise of EBF.

Studies show that better knowledge on infant and young child feeding may help mothers to practise EBF. Studies in Morogoro, Tanzania and in Zambia showed that women with inadequate knowledge on optimal breastfeeding practises, with perception of insufficient milk, and inadequate knowledge on breastfeeding problems tended to have poor EBF practise [15, 18]. In Kilimanjaro region, it has been reported that majority of women obtain knowledge on breastfeeding from health care providers through attending antenatal care clinics (ANC), postnatal and vaccination services [19]. The region has high coverage of attendance to ANC, post natal care and vaccination services, but the practise of EBF is still low. This shows the need for qualitative research that to give an in-depth understanding of dynamics that influence EBF practises.

Several quantitative studies have been conducted in Kilimanjaro region to assess factors associated with EBF practise. These studies reported similar findings to other studies in the country [14, 17]. The only published qualitative study on EBF was done among HIV positive mothers [20]. There are no qualitative studies that have been conducted to the general population to explore the factors that cause low practise of EBF. The qualitative methods in this study can help to answer critical questions about maternal behaviour, socio-cultural influences and practises that influence EBF practises. The aim of this study is to explore women’s knowledge, attitudes and practise towards exclusive breastfeeding in Kilimanjaro region. The information from this study may help in planning more effective interventions to improve the practise of EBF in this region.

## Methods

This study was conducted by focus group a discussion (FGDs) with mothers of infants aged.

0–12 months in three districts of Kilimanjaro region between August and October 2016. A total of nine FDGs were conducted, three in each district. In total, seventy eight mothers participated.

### Study areas and recruitment

The participating districts were Same, Rombo and Moshi Municipal Council. Moshi Municipal Council is the capital town for the region and it is more urban compared to Same and Rombo. The major ethnic group in Same is Pare and in Rombo is Chagga. Moshi Municipal Council has mixed ethnic groups.

The field research team comprised five people; two nutritionists, one nurse midwife and two medical doctors. Only four people were involved in running the FGDs. A nutritionist (female), first author of this article, moderated all the FGDs. Of the five, one was responsible with the logistics, two were field note takers, one was an observer and one moderator. The team did not introduce their professional background to the community in order for the mothers to open up on their breast feeding practises during the FGD.

In all three districts we recruited women from the community with the help of village executive officers and community health care workers. A list with mothers of infants’ aged 0–12 months was provided. Women were purposively selected from the list to represent a variety in age, parity and education. The selected women were contacted to join the FGDs on a set date. Of 100 women who were contacted for the study, 78 agreed to participate. Reasons for non-participation included the necessity to work on their farms or unavailability of child care while the mother participated in the FGDs. The inclusion criteria were women with infants aged 0–12 months who were still breastfeeding. We chose women with infants aged.

0–12 months because they were more likely to be able to recall their practises of exclusive breastfeeding and the challenges they faced. On the meeting day, objectives of the study were explained and mothers were asked to give a written consent to participate in the study. The FGDs were conducted in the ward offices, under the trees, in school or in a special room at the health centre. The meeting locations were chosen by the group members. The focus group comprised of 7–11 mothers and the discussions lasted for about one to one and half hours.

### Data collection

The FGD guide with written questions and probes was used during the discussions. The guide included questions on the meaning of infant and young child feeding practises and experiences towards exclusive breastfeeding, community influences, and practises regarding colostrum, insufficient milk, challenges and suggestions to improve the practise of exclusive breastfeeding in the community. The focus group discussions were conducted in Swahili by a trained nutritionist with experience in conducting FGDs. Confidentiality was discussed before each FGD and all participants were identified using numbers. To help clarify information, all discussions were tape recorded while other research assistants took field notes. Following each FDG was a debriefing in which the facilitator and three research assistants discussed new and important themes. Towards the end of the study, there was no new emerging information.

### Data analysis

Thematic analysis was performed according to five steps as described by Braun and Clarke (2006) [21]. The first step was familiarization of data in which the FGD scripts were transcribed verbatim, read and translated to English by the facilitators. The scripts were read and discussed by three authors. The discussion was also guided by the field notes. The second step involved development of initial codes by the first and last author. Priori codes were developed based on the FGD guide and the literature and grounded emergent codes were developed from the raw data in the transcripts. The authors discussed any emerging code, and after agreement the code was added to the list. The codes were developed independently and later compared by the authors. The authors discussed and refined codes until there was no other new code that emerged. The third step was searching for themes. A matrix table was used to list the codes. All codes that were related were sorted and listed into one comprehensive theme. The last two steps involved reviewing and refining themes and report writing.

## Results

In this paper the themes that were mentioned in all nine FGDs were breastfeeding knowledge, perceived benefits of EBF, reasons for not practising EBF, multiple sources for breastfeeding information and ways to improve EBF practise in the community. In each theme there were subthemes that are presented below.

### Socio-demographic characteristics of study participants

A total of 78 women participated in the study. The age of participating mothers ranged between19–47 years with mean age of 28 years. The majority, 65.4%had primary school education, 84.6% were married or cohabiting, 56.4% were farmers and 29.1% doing small scale business. Of the participants 46.2% were Chagas by tribe, 33.3% were Pares by tribe and 43.2% were residing in urban areas.The number of children for each mother ranged between 1 and 7 with an average of 3 children.

### Breastfeeding knowledge and perceived benefits

In all nine FGDs the mothers had quite good knowledge on EBF. They discussed the initiation of breastfeeding, the meaning of exclusive breastfeeding, continued breastfeeding and knew that the mother should practise exclusive breastfeeding for 6 months. They discussed the perceived benefits of EBF for the child and the mother. The most important reasons for practising EBF were: ‘it gives immunity to the child’, ‘promotes bonding’, ‘increases the child intelligent quotient’, ‘child grows faster’, ‘fills baby stomach’, ‘calms the baby’, ‘helps in family planning’, ‘mothers’ body returns to normal’, and ‘economical for the family’. The majority of mothers said they got this information from nurses/doctors when they visited ANC or came to clinic for vaccination of the child. Few mothers mentioned their past experiences as the reason for practising EBF.

The women also discussed that all children should be breastfed, exclusively or partly, up to 2 years or beyond, and that breastfeeding is the best thing the mother can give to the child. They admitted that this is what the community members know and all mothers have to abide to it.

Most mothers said that breastfeeding should be initiated directly after delivery to benefit the baby and stimulate milk production. Furthermore, they said that this early initiation of breastfeeding should be practised unless the mother has delivery complications or has undergone caesarean section. Finally, in all the nine FGDs, the mothers said that all infants should be given colostrums because the doctors and nurses tell them that colostrum is good for the immunity of the infant.

### Reasons for not practising EBF

Further discussions in the FGDs, revealed that many participants felt it was difficult to practise EBF the correct way. Some of the mothers said outright that though they know that EBF should be continued for 6 months, it is not practical to exclusively breastfeed for that long. Additionally, the mothers expressed challenges such as poverty, poor nutrition of the mother, need to return to work and cultural and social barriers that hinder the mothers from practising EBF.

### Poor nutrition

In the FGDs, the participants pointed out that proper nutrition during breastfeeding is crucial to practising EBF for the full 6 months. This was a challenge for the mothers, as most of them shared that they were not able to maintain proper diets. This caused them to experience low milk production which resulted in discontinuation of EBF. The mothers mentioned the types of food that they should eat to enhance milk production including *mtori (mashed bananas mixed with beef and soup)*, porridge, *kitawa (mashed bananas mixed with sour milk)*, and soups. Additionally, a few of the mothers mentioned that eating a balanced diet may help them to have more milk production.

### Resuming work

The majority of the participants worked in informal sectors such as farming and small scale business. Most mothers have the responsibility of feeding their families; hence they need to resume working soon after delivery. They said that they were typically working at the farmland ‘*shamba*’ for the whole day before returning home in the evening.

Excerpts from FGD discussions reveal the pressures that the mothers in the informal sectors face when returning to work that causes them to compromise EBF practises:

*“….We live in the village, we are supposed to go to the farm, fetch firewood, so it’s necessary to start giving porridge.This is because we leave our child in the care of someone at home, so when we are away what will the child eat? It’s difficult to give only breast milk all the time waiting for the child to reach six months” (FGD 3).*

*“…. I thought I am getting late to do my other stuffs ‘farming and small business’ so I decided to give porridge when the child was four months” (FGD 9).*

Mothers working in the formal sector shared similar challenges about how their work interfered with the practise of EBF:

“*Maternity leave is only three months, how can we practise EBF for six months?” (FGD 2).*

Currently, the Tanzanian national policy allows for mothers to breastfeed 2 hours per day until the baby is 6 months of age, after completion of maternity leave. However, in some situations, the mothers who were working in a formal sector shared that they were denied the 2 hours for breastfeeding.

*“I was not given the two hours to allow me to go and breastfeed, after the maternity leave of three months… Moreover, the employer wanted me to work from 8:00am to 6:00pm after the three months of maternity leave….” (FGD 1).*

Some mothers had to quit work after the maternity leave. Those who kept their jobs mentioned that the only choice to feed the child while away from home is to start giving cow’s milk, thin porridge, *kitawa* or *mtori*. In many cases, these alternative foods were introduced into the child’s diet before 3 months of age so that the child should be used to these foods before the mother returns to work.

The mothers mentioned that they do not receive enough support from their partners/spouses or other family members to practise EBF for 6 months. Staying at home to breastfeed is seen as laziness by others. Since most women are the sole providers/bread earners and are responsible for the food in the household, other children in the family may suffer from hunger if the mother does not return to work.

### Inadequate knowledge on expressing breast milk

The majority of the mothers admitted that they had inadequate knowledge on milk expression, which made them give alternative foods to breast milk when resuming their work.

*“We have been told that if you are going to work you can express breast milk and keep it for three hours. After that time the milk will no longer be good for the baby. But we don’t know the details of this and we have not thought of trying it to our babies” (FGD, 2).*

Additionally, few mothers said they have heard about breast milk expression, but they were not sure how long the expressed milk can stay fresh for the child. Others knew about breast milk expression as a solution to prevent breast engorgement, but were not aware that the expressed breast milk could be used to feed the child.

### Crying baby linked to perception of insufficient milk supply

In all nine groups, the mothers said that the baby crying was a big challenge because it made some mothers stop practising EBF before the child reached 6 months of age. The mothers shared that they associated crying with hunger or thirst for the child. They said*, “Babies cry because they are hungry, breast milk is not sufficient”.* Giving water, thin porridge, or roasted banana has been a traditional practise. The tradition of giving water early (within the first week after delivery) has made mothers believe that EBF practise include giving water to the child. The thin porridge is prepared by cooking porridge and sieving it by adding water until it is as light as milk. Traditionally, it is thought that the child can start drinking thin porridge at 1 month of age. However, the participating mothers were unsure if giving water or porridge is the solution to stop the child from crying.

*“Though the nurse tells us that we should breastfeed exclusively for six months, in reality it is very difficult. We cannot practise EBF without giving water. At least the nurse should tell us to EBF and give water…. this is what everyone in the community knows….just think how can the child survive without drinking water?”(FGD 2).*

*“I understand that the child should be breastfed for six months without mixing with other foods but the problem is, the child cries a lot... I know my milk is very light and cannot satisfy her”( FGD 5).*

*“If I go to the clinic and the child has not gained weight, the nurse yells at me...... and also my baby cries a lot I think my breast milk is insufficient...” (FGD 2).*

*“We give water or porridge but we don’t really know what the child wants, we think giving them water or porridge is the way of calming them down when they are crying, though it has never helped” (FGD 6).*

### Multiple sources of EBFinformation

The participants reported to have received information about breastfeeding from multiple sources such as; health care providers, mothers, aunties, sisters, friends and neighbours.

### Advice on exclusive breastfeeding from health care providers

The majority of mothers said they received advice on breastfeeding when attending the antenatal care clinic, during delivery or when coming for vaccinations. Only a few mothers stated that they had never received advice from health care providers from the nearby health centres. The information received from the health care workers included: giving colostrum, early initiation of breastfeeding and practise EBF for 6 months, handling breastfeeding problems, correct positioning and attachment, benefits of breastfeeding and breastfeeding advice if the mother is HIV positive.

Some mothers said that health care providers had given them advice to give water or infant formula immediately following delivery if breast milk flow is delayed or the baby starts crying.

### Advice from close relatives

The mothers also stated that they had received advice from their mothers, other relatives, including in-laws or friends. They mentioned that the advice from their mothers or in-laws was based on their experience from long ago. The kind of advice commonly known in the community focused on handling a crying baby, giving water early to quench child’s thirst, giving food early to prepare the child’s stomach (food given included roasted banana with butter, *mtori, kitawa* or thin porridge), and give herbs to relieve abdominal pain called “*chango*”.

The following are examples of some quotes by mothers;

*“*…*If the child is crying, our mothers or mothers in law would tell us to give the child water or food” (FGD 4).*

*“…We normally give water in the first week and porridge when the child is three weeks old” (FGD 9).*

*“…The reason for giving water or food is to prepare the child’s stomach so that he/she cannot be stubborn to eat other foods later and to stop the child from crying”(FDG 8).*

### Navigating between different types of advice

The groups discussed the contradiction between the information received from health care providers and other community members (mothers, mothers-in law, aunties, friends and sisters). The participants followed a certain advice in order to handle difficulties during breastfeeding. The choice of advice depended on the mother’s trust of the person giving the advice. Many mothers trusted the experiences of their mothers,in-law and friends. This is because the advice comes from those who have personal experience in child feeding. The discussions also revealed that EBF practise may be influenced by power relations in the extended family. Many mothers spoke on the difficulty to adhere to advice from health care providers because of fear of abiding to their mothers or mother-in-laws wishes.

*“Not abiding to what they say, you will be told you don’t respect elders, so if you don’t like to quarrel you do what they say” (FGD2).*

Few mothers opted to choose the advice from health care providers.

*“When I delivered this child, my mother in law told me to give water, and I did not agree with her advice, as I understand that a breast is like a sieve; it knows the time to give water and food, so this helps to quench the child’s thirst”(FGD 4).*

Other mothers opted to follow the advice that they got from the health care provider after experiencing challenges of illness. *“….. I experienced that my children were getting sick very often and the doctor told me: mama have you given anything apart from breast milk and I said yes. He told me that was the cause (of the child’s sickness). So for the present child I said I will do EBF to avoid those problems” (FGD 9).*

### Ways to improve EBF practise

#### Breastfeeding education

The majority of the mothers stated that breastfeeding education to their close relatives will help them practise EBF for 6 months. The mothers were concerned about getting updates on breastfeeding when they visit clinics for vaccination. They believe more information will help in practising EBF. They also thought that other family members who have influence on the practise of EBF should be informed about the benefits of EBF to support mothers in breastfeeding.

*“In our community we still need more information regarding breastfeeding. Our elders and other people in the community should be well informed about exclusive breastfeeding. It is very difficult to practise what the nurses have told you if an older woman is there and she tells you how to feed your child.”(FGD 2).*

However, during the discussion many mothers were uncertain if other influential family members would be willing to attend trainings in EBF.

#### Use of peers to educate mothers

Another suggestion was to institute women with prior experience in proper EBF as counsellors to aid mothers in these practises. Mothers who have practised EBF successfully could relay their own personal experiences, which may encourage mothers to practise EBF.

#### Longer maternity leave and support for breastfeeding mothers

Those mothers who were formally employed were concerned about extending the maternity leave so that women are able to practise EBF. Those in informal sectors mostly needed support from their partners or other family members. Receiving help may allow mothers to stay at home and correctly practise breastfeeding.

## Discussion

The mothers in this study had overall good knowledge of EBF, including the benefits of colostrum. However, they were not able to practise EBF in their real lives for several reasons. The reason for not practising EBF included poor perceived maternal nutrition, pressure for women to return to work, inadequate knowledge about properly expressing breast milk and perceived insufficiency of milk supply. This study provides a unique window into breastfeeding knowledge as well as the attitudes and practises of Tanzanian mothers in the twenty-first century. The gaps between knowledge and practise, the tensions between advice from the health sector and that of respected family members and the strong belief that babies cannot live without water, have all interfered with EBF practise.

In this study mothers did not discard colostrum and it was considered to have protective effect for the child’s health. The majority of mothers were able to initiate breastfeeding within 1 hour after delivery. Giving colostrum and initiating breastfeeding within 1 hour aligns with the WHO recommendations [1]. These findings are contrary to those of earlier studies in Tanzania, where it was reported that women were discarding colostrum out of fear that it is dirty milk [15]. However, the results from the present study indicate a gap between knowledge and practise, since only a few mothers were able to practise EBF for 6 months. Many mothers believed that giving water was a part of EBF practise and helped to quench the infant’s thirst. This practise has been reported by other researchers in different settings [22, 23]. A study in Kigoma showed that mothers had good knowledge in EBF, but few were able to practise it [16]. Intervention messages that breast milk is sufficient to quench child’s thirst might be helpful to improve the practise of EBF.

Early introduction of complementary foods was reported in all groups. The most common foods and drinks given to infants were thin porridge, *kitawa* (mashed banana with sour milk)*, mtori* (mashed banana with beef) and roasted banana with butter. Fear of insufficient milk, the baby not gaining weight and baby crying were the reasons for giving foods or liquids, as baby crying was perceived as a sign of hunger. In addition, foods were given early to prepare the child’s stomach and prevent stubbornness when the mother had to be away for work and other family members needed to feed the baby. Other researchers have reported perceived insufficient milk as a barrier to practise EBF [23–27]. The practise of giving liquids or solids other than breast milk before 6 months does not follow the WHO recommendations for infant feeding [3]. It poses greater risks to child health; the child digestive system is not well matured to start digesting other foods. Particularly, in developing countries where there is limited access to clean and safe water, infant’s are at high risk of infections, malnutrition and death [2, 28–31]. There is a need to have behaviour interventions to practise EBF and with an emphasis on the physiology of milk production and signs of hunger. This may help mothers understand that breast milk is enough for the infant.

Many women in the present study received differing advice regarding breastfeeding practises. While the information on the benefits of EBF was taught by the health care providers, the majority of mothers did not practise EBF. How women internalize the advice given by health care providers has been reported by other researchers in Tanzania (Morogoro), Vietnam and Myanmar [15, 22, 32]. Women in the present study tended to believe what they had been taught by their close relatives, since they have more experience regarding child feeding. Close relatives have been reported by other researchers to have a strong influence on child feeding practises [18, 24, 33]. Thus, there is a need that close relatives and key community gate keepers understand the recommended child feeding advice and help to support breastfeeding mothers to practise EBF.

Resuming work after a short maternity leave prevented many mothers from practising EBF. Other studies have reported that resuming work early and short maternity leave conflicted with EBF practise [26, 34]. The majority of mothers in the present study were the bread earners/sole providers of their families. Many reported to lack support from their spouses to practise EBF. Thus, the mothers opted to give their children complementary foods so they could go to work. The literature shows that family support is an important factor for successful breastfeeding [35]. Interventions targeting partner/spouses and employers to support mothers when they are breastfeeding may help to increase EBF practise.

One of the steps of BFHI is to help mothers’ breastfeed while away from home by expressing breast milk [36]. This can be achieved if mothers have proper knowledge on milk expression and storage of the milk. The results of this study suggest that mothers have inadequate knowledge on milk expression; they believed it should only be practised to prevent breast engorgement. There is a need for more emphasis on the education of mothers on milk expression to help mothers’ when away from home.

Poor nutrition was also reported to be a major barrier to the practise of EBF. Mothers said that not eating fluid foods like *mtori, kitawa* and soups after delivery led to poor milk production. A study by Amir and colleague showed that improvements in maternal diet increased the odds of practising breastfeeding [37]. The mothers’ thoughts of poor nutrition may lead to stressful condition, and hence interfere with the process of milk production. Nutritional counselling in breastfeeding mothers during ANC visit is important to help mothers practise EBF.

### Strength and limitations of the study

The strength of the present study is that large varieties of women from diversified cultures, and from both rural and urban areas, were recruited from the communities. They gave a rich and in depth understanding of breastfeeding practises in these communities. Social desirability bias could be the limitation to the study as some mothers might have withheld what they thought to be negative aspects of their breastfeeding practises. Furthermore, we conducted one FGD at the special room nearby the health centre; this could have increased the social desirability bias. To minimize bias, the discussions were moderated by a person who knows the Swahili language and is competent in the field of child feeding. In addition, health care providers working in the health centers were not involved in the FGDs.

## Conclusion

The results from this study indicate that mothers had adequate knowledge on EBF despite the misconception that giving water is part of the EBF practise. Thus, the campaigns messages promoting EBF have reached mothers. However, this knowledge has not been translated into practise. Many different challenges that prevented the mothers from practising EBF were voiced during the discussions. These included poor nutrition for the mother, baby crying, fear of insufficient milk, early resumption of work and influence from close relatives. Knowledge of these factors might be helpful in designing future interventions to promote EBF.The following recommendations may help to improve EBF practise in the community;

- Targeted intervention and education aimed at mothers and the community to promote breast milk as more than adequate to quench thirst and hunger.
- Mothers should be taught how to express and store breast milk, how to respond to their infants hunger cues at antenatal, post-natal and follow up visits.
- Close relatives and employers should support breastfeeding mothers. Close relatives should be involved on breastfeeding targeted intervention for them to acquire the right knowledge on infant feeding.

## Abbreviations

ANC: Antenatal care
BFHI: Baby friendly hospital initiative
EBF: Exclusive breastfeeding
FGD: Focus group discussion
IYCF: Infant and young child feeding
pMTCT: Prevention of mother to child
SSA: Sub-Saharan Africa
